# Lifecycle HTA: promising applications and a framework for implementation. An HTAi Global Policy Forum Task Force report

**DOI:** 10.1017/S0266462324000187

**Published:** 2024-05-10

**Authors:** Franz B. Pichler, Meindert Boysen, Nicole Mittmann, Ramiro Gilardino, Andrew Bruce, Kenneth Bond, Rick A. Vreman, Nathalie Largeron, Judit Banhazi, Daniel A. Ollendorf, Mohit Jain, Sheela Upadhyaya, Wim G. Goettsch

**Affiliations:** 1 Confluence Health Consulting, Sydney, NSW, Australia; 2 National Institute for Health and Care Excellence (NICE), London, UK; 3 Canadian Agency for Drugs and Technologies in Healthcare, Ottawa, ON, Canada; 4 MSD, Zurich, Switzerland; 5 Amgen, Sydney, NSW, Australia; 6 Institute of Health Economics, Edmonton, AB, Canada; 7 Roche, Utrecht, Netherlands; 8 Sanofi, Paris, France; 9 Menarini, Zurich, Switzerland; 10 Tufts Medical Center, Institute for Clinical Research and Health Policy Studies, Boston, MA, USA; 11 BioMarin, London, UK; 12 Life Sciences Consultant, London, UK; 13 Zorginstituut Nederland (ZIN), Utrecht, Netherlands

**Keywords:** HTA policy, Health technology assessment, Technology lifecycle, Life cycle HTA, Uncertainty, HTA reassessment

## Abstract

The 2022 Health Technology Assessment International (HTAi) Global Policy Forum (GPF) established the goal of developing a position statement and framework for lifecycle HTA (LC-HTA), through a Task Force leveraging multi-stakeholder monthly discussions and GPF member input. The Task Force developed a working definition: LC-HTA is a systematic process utilizing sequential HTA activities to inform decision making where the evidence base, the health technology itself, or the context in which it is applied, has a potential to meaningfully change at different points in its LC. Four key scenarios were identified where it was considered that an LC-HTA approach would add sufficient value to HTA bodies and their key stakeholders to justify the additional resource burden. Based on the four scenarios, a high-level LC-HTA framework was developed consisting of (i) defining the decision problem, (ii) sequencing of HTA activities, and (iii) developing optimization criteria. Subsequently, the Task Force developed operationalization guidance for LC-HTA in a companion paper.

## Introduction

An outcome of the 2022 Health Technology Assessment International (HTAi) Global Policy Forum (GPF) was the recommendation to establish a multistakeholder task force to build on the Forum’s discussion about the lifecycle (LC) approaches in Health Technology Assessment (HTA) (1). The objective of the Task Force was to develop a position statement, including developing a definition of lifecycle HTA (LC-HTA), identifying where LC-HTA approaches could add value to HTA bodies and HTA-related stakeholders, developing an LC-HTA framework and high-level guidance for how to operationalize LC-HTA. The scope of this position statement was primarily focused on the application of LC-HTA to individual technologies (drugs, devices, digital health, and surgical interventions); however, we recognized that LC-HTA might additionally have value in terms of multiple technologies, including treatment sequencing and supporting guideline development.

The Task Force was composed of a geographically diverse group of GPF members and people representing HTA bodies, academia, technology developers (pharmaceuticals and devices), and nonprofit organizations. The Task Force was guided by a chair (M.B.) and cochairs (N.M., R.G., and A.B.) and supported by a writer (F.P.). The Task Force met monthly to develop the position paper, through consensus. Intermediate drafts were presented for review and feedback to the GPF at their March 2023 and June 2023 meetings. Additional feedback was solicited from members of the broader HTAi community. The manuscripts were reviewed by HTAi’s Scientific Development and Capacity Building Committee.

Two companion papers were developed to describe and address the challenges associated with LC-HTA described above. This paper focuses on the strategic reasons why LC-HTA would interest HTA bodies and the second focuses on operationalizing LC-HTA. This first paper advances an argument for why HTA bodies might want to use LC-HTA, defines LC-HTA, describes scenarios where LC-HTA might be of greatest value, and provides a framework for how LC-HTA approaches can be structured.

## The concept of lifecycle in HTA

While the concept of evaluating technologies across their LC is not new, with discussion in both regulatory (2) and HTA (3;4) contexts, the HTA community is increasingly discussing how to apply LC approaches in HTA (1;5). HTA is defined as “… a multidisciplinary process that uses explicit methods *to determine the value of a health technology at different points in its lifecycle.* The purpose is to inform decision-making in order to promote an equitable, efficient, and high-quality health system (6).” The definition indicates the potential need to consider a LC approach during HTA.

There is a growing recognition that HTA systems need to adopt an LC approach to respond to the need for value assessment across the life span of health technologies (1;7-12). For example, concern has been raised that changes in the evidence base or clinical pathway might invalidate initial HTA decisions (11) or require updated HTA guidance to inform downstream stakeholders (1). This concern may be particularly relevant where there is high initial evidentiary uncertainty or decision-making risk, for example, related to reimbursement (8;9). It has also been argued that using HTA to routinely assess technologies across their LCs could increase efficiency and equity in managing health resources (10).

LC-HTA has been proposed as a way to manage evidentiary uncertainty (5); address changes in the evidence base, the design of the technology, or the clinical pathway (1;11); and support iterative decision making (7;10). Despite different applications, these proposed approaches all include well-established components of standard HTA that are applied to varying phases of the technology LC to address specific decision problems (1;7;11). An LC approach may represent a prospectively planned systematic sequencing of such components (11) and may also include some forms of trigger (7;8;11), leading to an HTA reassessment. However, there is no consensus definition of the term “LC-HTA” (1) or how this term relates to similar concepts such as Health Technology Reassessment (13), “Living HTA” (8;9), or “Health Technology Management” (10). As in other areas of HTA, a consensus definition would facilitate communication and collaborative action among the diverse stakeholders concerned with developing LC approaches.

Differences among HTA agencies also raise questions about the feasibility of implementing LC-HTA approaches. The varying remits and capacity of many HTA bodies result in the prioritization of single, comprehensive HTA reviews of new technologies following marketing authorization (10;14). There is also concern by HTA bodies about the feasibility of implementing LC-HTA given the additional resource demands that such an activity would entail (1). For this reason, the HTAi GPF also recommended a need to identify and define the decision problems where LC-HTA approaches can add meaningful value (1;8;11). LC-HTA approaches will likely require selective implementation for most HTA bodies and will need to consider both constraints for the HTA body and the potential of the approach to add meaningful value in addressing the decision problem.

## Goals of the position paper

This position paper sets out to:

1. define why the HTA community would want to consider LC-HTA approaches;
2. provide a definition for LC-HTA;
3. describe the health system challenges where LC-HTA might offer the greatest opportunities; and
4. develop a framework to conceptualize how LC-HTA approaches might be structured.

## Why the HTA community would want to consider LC-HTA approaches

A key driver for why HTA stakeholders are showing interest in LC-HTA approaches relates to the core purpose of HTA. Despite significant diversity in the remit and application of HTA bodies around the globe, the purpose common to all HTA is to inform decision making through the assessment of the value of a health technology (6). As such, there is an underlying principle that is also common to HTA doers, which is to reduce and manage decision-making uncertainty to enable the timely and evidence-based assessment, appraisal, adoption, utilization, and management of technologies in healthcare. HTA can improve the quality of the information for decision making through its processes and methodologies, and by improving the quality of the evidence base, as HTA bodies seek to do through guidance documents and activities such as providing scientific advice. Therefore, interest in LC-HTA approaches is a natural extension of the core purpose of HTA and is needed to inform decision making about the changing value of a technology at different points in its LC (e.g., 15).

To an extent, HTA bodies already have mechanisms that can provide information regarding the changing value of a technology via activities across the LC or through special pathways. Pre- and post-launch activities routinely conducted by HTA bodies or other organizations, such as horizon scanning, early dialogue/scientific advice, managed entry agreements, monitoring implementation, health technology reassessment, optimization, and disinvestment, may be considered potential elements of an LC approach (1). Part of the motivation for HTA bodies to be interested in a more structured LC-HTA approach is that such activities are not always coordinated and may be undertaken by different organizations or groups within and outside of an organization.

The development of an LC-HTA approach can draw lessons from past experiences and recommendations concerning coordination across a sequence of activities and across stakeholder groups. The 2018 review of global horizon scanning activities by the HTAi GPF (16) recommended an LC approach for the purpose of improving the integration of horizon scanning with downstream decision making. Learnings from the Dutch and UK implementation of managed entry agreements (MEA), through the Conditional Financing (CF) and the Highly Specialized Technology (HST) programs, respectively, suggest that some of the key challenges in MEA can be mitigated through preplanning and coordinated data collection, the need for ongoing stakeholder consultation to align expectations and prevent discrepancies between initial agreements and final data at reassessment, and to ensure a strong mechanism for incorporating new evidence into decision making (17-19).

Another motivation for HTA bodies is to consider how to address emerging challenges to existing HTA approaches that are arising from rapid technological advancements. For example, the new German regulatory and reimbursement pathway for digital healthcare applications (DiGA) will require adaptation of their current HTA processes in order to account for emerging evidence both as a consequence of limited evidence at the time of product approval and resulting from ongoing product modification (20).

## Definition of LC-HTA

LC-HTA is characterized by two features that differentiate this concept from other HTA activities: (i) it explicitly addresses change over time, and (ii) it connects and coordinates several distinct HTA activities. While the definition of HTA states that the value of a health technology is determined at different points in its LC (6), this does not mean that all HTA activities are iterative. In practice, most HTA activities across the technology LC represent snapshots (8) taken to inform a decision at a single point in time; this includes activities such as horizon scanning, HTA of a technology for market entry, or health technology reassessment of a technology currently in use. By contrast, the purpose of LC-HTA is to manage change over time, whether that relates to an evolving evidence base or a changing clinical context. LC-HTA activities can begin early in the LC of a technology, for example, to inform decisions about the development of the evidence base, or in later phases, such as determining whether change is sufficiently meaningful to require an HTA reassessment. We note that it will be important to follow a deliberative process (21) to ensure a common understanding among those involved, for example, in defining a threshold for what would constitute meaningful change. The other differentiator relates to the connection of HTA activities. Although closely linked, many HTA activities are standalone; for example, the use of HTA by an HTA body for an initial reimbursement recommendation is not always reliant on information from horizon scanning. LC-HTA implies interconnected, sequential HTA activities that require prospective and systematic planning. Considering these two differentiating features, we propose the following definition: LC-HTA is a systematic process utilizing sequential HTA activities to inform decision making where the evidence base, the health technology itself, or the context in which it is applied, has a potential to meaningfully change at different points in its LC.

## Application of LC-HTA

Our definition of LC-HTA implies that there is a broad range of decision problems facing HTA bodies where such an approach could be applied. We have taken the perspective of the prospective development of an LC-HTA pathway by an HTA body.

Some HTA bodies have established special pathways related to specific decision problems that could be understood as forms of LC-HTA. An example of an LC-HTA approach that occurs early in a technology LC is the Early Value Assessment (EVA) program for medical devices that has been developed by NICE. This program includes a sequence of HTA activities that identify and prioritize key areas of unmet need in the U.K. health system and identifies promising technologies in early development. NICE proactively engages with the technology developers with the intention of providing development guidance, support with data collection, and early access to the health system (22). An example of an LC-HTA approach for more mature technologies is the early access authorization (EAA) program managed by France’s Haute Autorité de Santé (HAS). This program is designed to give patients prompt access to emerging therapies before a regulatory authorization or final reimbursement decision (23).

## Scenarios where LC-HTA may be applicable

We consider four key scenarios where applying an LC-HTA approach could yield sufficient value to HTA stakeholders to justify the additional resources required. We conceptualize these scenarios as high-level challenges for HTA bodies that may stem from a variety of different decision problems (see Table 1).

1. Uncertainty relating to limited evidence at the time of review. Although uncertainty related to limited evidence is a relatively common criticism by HTA bodies, there are situations where the extent or context of this evidentiary uncertainty is sufficiently meaningful that an LC-HTA could be warranted. One example is where the initial evidentiary package is limited because of an accelerated regulatory approval of a technology based on promising, early data in situations of high unmet need, such as rare diseases. Another example of limited evidence relates to lengthy time horizons for evidentiary uncertainty to be resolved, such as gene therapies where the intervention’s ongoing impact, safety, and durability are unknown.
2. Technology may be modified over its **LC**. We consider LC-HTA to have potential utility where the technology itself is not static but can change over time to an extent where there would be a meaningful difference if an HTA reassessment were undertaken. Examples of such change could include medical device “incremental innovation” where the technology product design is periodically upgraded. Another example of a more dynamic form of change relates to changes to diagnostic gene panels through either the inclusion of additional markers or a change in the scientific understanding of existing markers. A more extreme version of such innovation would be technologies that change constantly, such as using Artificial Intelligence in health care.
3. A learning curve related to utilizing technology in practice changes its outcomes. The outcomes delivered by an intervention may change through clinician experience and real-world practice. Effectiveness and safety may change as practitioners gain experience with a complex intervention, such as with a surgical robot. In addition, clinical experience over time may change how medical interventions are used in practice. As real-world utilization provides an increased understanding of how an intervention performs in the context of patient diversity and the local health system, clinicians can optimize their utilization of the technology, for example, changing in dose or timing. This can even extend beyond the original regulatory label. For example, in oncology, new pharmacological interventions are often approved using clinical studies on late-stage patients but, once available to clinicians, may become used in earlier-stage patients.
4. Health service context impacts or is changed by the technology. LC-HTA may also add value where the technology impacts or is affected by changes in the context in which the technology is situated. For example, where a technology causes a significant disruption to existing care pathways, there may be value in a reassessment sometime after implementation to review and evaluate the outcomes of that disruption. Where the context changes independent of the technology, such as a change in the care pathway or policy changes related to HTA methodologies or decision-making parameters (for example, where an HTA methodology guidance changes to allow a form of evidence previously not accepted), then an LC-HTA may be of use to steer the development of technology in anticipation of upcoming policy changes or to assess technologies in a care pathway that has been subject to change. This scenario demonstrates that LC-HTA can have applications beyond individual technology assessment, such as a multi-technology appraisal, disinvestment decision making, or guideline development/update.

**Table 1.** Scenarios where lifecycle approaches might add greatest value

Scenarios	Why this is a challenge for traditional HTA	Example product areas	Application of LC-HTA
**1. Initial information about the technology is limited**			
Limited information on efficacy and safety	The information about the health technology is limited due to small trial sizes or other constraints that result in high clinical uncertainty at the time of review.	Rare diseases, medical devices, surgical procedures, RCTs where blinding is not possible, pediatric populations, gene therapies	High
Accelerated regulatory approval	Regulatory approval based on early, promising data for high unmet need patient populations presents less comprehensive information for HTA than under standard regulatory approaches.	Breakthrough pharmaceuticals	High
Long–term effects based on surrogates	The health technology uses surrogate data or models to predict key long–term efficacy as trial lengths to obtain such data are impractical. The surrogate end point will get validated (or invalidated) with time.	Disease modification of long–term progressive diseases, e.g., Alzheimer’s disease, some rare diseases	Medium
Long–term safety and durability effects are unknown	Where the intervention will have an ongoing impact, such as over the patient’s lifetime, and long–term effects, especially the intervention’s safety and durability, are unknown.	Advanced medical technology products (AMTPs), regenerative medicines, rare diseases	High
**2. The individual technology can alter its effectiveness/safety or other relevant aspect over its lifecycle**			
Incremental innovation	The health technology is not static; it can change and evolve to improve on weaknesses or add new features (“upgrades”). Improvements are directed, and hence each version is typically expected to be a stepwise improvement in some aspect compared to the preceding version.	Medical devices, cell and gene technologies	High
Dynamic innovation	Similar to incremental innovation but at a faster pace due to rapidly expanding knowledge and less predictable than a version upgrade as it reflects the expanding scientific understanding of the field.	Genomic diagnostics, digital therapeutics, and predictive medicines	Medium–High
Fluid innovation	Constantly evolving technology that, without regulated control of the algorithm, is neither directed in its evolution nor at any point is sufficiently “static” to allow a full HTA.	Artificial Intelligence	Medium
**3. Learning curve related to the utilization of the technology in practice changes its outcomes**			
Outcomes are related to the experience of use	For complex interventions, optimal effectiveness, safety, and appropriateness of use require training and clinical experience.	Medical devices, surgical interventions	Medium–Low
Lack of comparative effectiveness information	Where the intervention lacks comparative evidence versus a key comparator relevant to the local health system at the time of the initial assessment but through utilization, such evidence becomes available.	Products for which postlaunch RWE is critical	Medium
Optimizing the use of the technology	Clinical experience using medical interventions on the margins of their label may refine their use, including improving outcomes, via changing dose, timing, or use in combination; or broadening the use beyond the indication.	Life–saving medications, typically oncology	High
**4. Health service/delivery context impacts or is changed by the technology**			
Highly disruptive technology	Where uptake of the technology promises to disrupt the existing care pathway, perhaps requiring a significant investment or disinvestment of facilities relating to the pathway components being displaced	PET scanners, AMTPs	Medium–low
Change in the clinical context	When there is a significant change in the clinical context expected, such as a new understanding of the disease (e.g., identification of disease subtypes) and/or guideline changes that alter the care pathway.	Shift to histology independent oncolytic; diagnostic switch from PAP smear to PCR testing.	High
Policy changes	Policy changes related to the HTA body processes or methodologies result in the ability to utilize new forms of evidence (e.g., RWE, basket trials, patient input, etc.) or change decision–making parameters.	Previously non–approvable technologies based on their evidence (basket trials, complex analyses); or refinement of existing reviews based on new methodological approaches.	Medium

*Note*: Table 1 presents the four key scenarios where the TF believed that an LC-HTA approach has the potential to make a meaningful difference in addressing a key challenge for traditional HTA. Example decision problems are provided for each scenario in order to show the range of challenges and decision problems that might arise and to indicate that, even within these scenarios, there is variability in the ability of LC-HTA to resolve the challenges. The column “Application of LC-HTA” represents a qualitative assessment by the TF of the ability of an LC-HTA process to address the decision problem in the specific example. Scoring: Low (limited benefit); medium (some benefit, but other processes required); high (likely to resolve the issue). There may be decision problems that are represented by multiple rows. In such instances, the application of LC-HTA to cross-cutting problems may be equally or more beneficial compared to assessing individual rows separately.

## A framework for LC-HTA

The breadth of potential decision problems within the four scenarios demonstrates that LC-HTA has a wide range of applications. This led the Task Force to conclude that rather than a “one-size-fits-all” pathway for LC-HTA, implementation will require tailoring to the decision problem. This observation led the Task Force to develop an LC-HTA framework with three key components that can be used to describe an LC-HTA process.

1. *Defining the decision problem*: Develop a clear decision problem to be used to guide where and why in the technology LC to apply LC-HTA and for what outcome. A key element is identifying whether addressing the decision problem through the additional activity will be sufficiently meaningful to justify the resources spent.
2. *Sequencing of HTA activities*: To resolve the decision problem, it will be necessary to determine which HTA activities are required and how they should be connected to ensure appropriate alignment, coordination, and predictability.
3. *Developing optimization criteria*: Development of clear criteria or guidelines to determine eligibility to an LC-HTA process or when a specific step in LC-HTA should be activated to ensure optimal utilization of different steps in the process. An important implication of utilizing optimization criteria, from an efficiency perspective, is a transparent process to determine when certain HTA activities are worthwhile and when they are not. For example, prospectively planned optimization criteria for an LC-HTA process designed to address changes in surgical robot software could help ensure that HTA reassessment is only activated if a software upgrade changes the technology’s effectiveness or safety profile to a sufficient extent where the original HTA decision might be invalidated.

The LC-HTA framework is intended to be useful for describing real-world implementation of LC-HTA approaches and to help structure the development of new approaches. An important additional aspect for this framework will be to decide which stakeholders to involve and for what components to ensure appropriate alignment, coordination, and predictability. Task Force recommends utilizing deliberative processes (21) and broad stakeholder involvement (1) is an important consideration for each of the three components of the framework.

With respect to describing existing approaches, we utilized the framework to characterize the HAS EAA program (Table 2) and the U.K. EVA scheme (Table 3). A standardized approach, such as the LC-HTA framework, will support comparison between potentially diverse applications of LC-HTA, demonstrate which aspects might be missing in existing pathways, and offers a way in which to structure the operationalization of new LC-HTA approaches.

**Table 2.** How the LC-HTA framework would characterize the HAS Early Access Authorization Scheme

Framework	Characterization of AAP pre–MA pathway
The decision problem	How to enable early access to promising therapies for patients with high unmet needs that lack alternative treatment options, and where it is undesirable to delay treatment until after the lengthy regulatory and reimbursement processes.
Sequencing of HTA activities	The manufacturer makes an application for the pathway with an abbreviated dossier. If eligible, HAS undertakes a “light HTA assessment” of the clinical evidence available and the manufacturer’s development plan. A time–limited AAP is granted with conditions of compliance to a protocol for therapeutic use in a defined population, including collecting data from all patients treated and periodic reporting of these data. The manufacturer is expected to contribute to the resourcing required for data collection. On completion of regulatory approval, a final, full HTA review is conducted that can lead to conversion to a standard reimbursement model.
Optimization criteria	The AAP scheme includes three optimization criteria: Gateway criteria into the scheme to ensure that only products that meet the requirements are accepted. Predefined time points for reporting and review of RWD, which is necessary for the scheme renewal. A trigger of regulatory approval that initiates the comprehensive HTA review.

*Note*: The Early Access to Medicines (AAP) scheme (23) administered by the French HTA agency, Haute Autorité de Santé (HAS), is an example of an LC-HTA approach in action. This scheme has two pathways: prior to regulatory authorization (pre-MA) and prior to reimbursement (post-MA). This example focuses on the pre-MA pathway.

**Table 3.** How the LC-HTA framework would characterize the NICE Early Value Assessment (EVA) Scheme

Framework	Characterization of AAP pre–MA pathway
The decision problem	How to provide early access to promising technologies in development that have the potential to address prioritized areas of unmet need in the UK’s health and social care system and for which the evidence base is not yet complete.
Sequencing of HTA activities	NICE utilizes a process termed “topic intelligence” (a form of horizon scanning) to proactively identify priority areas in the health and social care system, followed by identifying emerging technologies that may address the prioritized areas. NICE proactively engages with manufacturers of the identified technologies to invite them into the scheme. The next step in the process is an early value assessment that includes developing an evidence–generation plan for technologies deemed suitable for early patient access. NICE aims to provide opportunities for technology developers to work with key stakeholders who can help deliver the evidence–generation plan. Following the delivery of the evidence, a standard NICE appraisal will be undertaken.
Optimization criteria	The EVA approach includes two main optimization steps Initial eligibility criteria involve identification and prioritization of the health system needs and suitable technologies that have the potential to address those needs. Prior to granting early access, an early clinical and economic assessment is used to determine if a technology can progress further in the scheme.

*Note*: UK’s NICE has developed the EVA approach (24) for the purpose of identifying, guiding development, and providing early access to, immature technologies that have been identified as having the potential to address key health system priorities. The approach is applicable to medical devices, diagnostics, and digital products.

## Conclusion

The Task Force believes that HTA bodies can implement LC-HTA approaches to efficiently and effectively address a range of decision problems representing challenges for traditional HTA processes. Implementation of LC-HTA will require a degree of agility within HTA organizations to develop new pathways that encourage linkage between discrete HTA activities and collaboration with various stakeholders. While HTA bodies are likely to be the organizations that advocate for the adoption of LC-HTA in their jurisdictions this does not imply that these bodies will be doing all of the work. Depending on the local circumstances, some of the HTA-related activities utilized by an LC-HTA approach may be undertaken by other parties (e.g., an horizon scanning unit, clinicians, the manufacturer, etc.) and therefore LC-HTA is likely to require greater alignment across stakeholders in the HTA ecosystem.

Successful use of LC-HTA may provide HTA bodies with a means to adapt to many of the emerging challenges related to the rapidly evolving health technology environment. We look forward to feedback and comment from others who might have found other scenarios in which LC-HTA may provide a unique solution to the challenge of changing healthcare technologies and system needs. The three components of the framework are a starting point and may be developed further based on new insights.

The conclusion of this paper leads naturally to the question of how HTA bodies might operationalize LC-HTA. The companion paper in this journal (24) discusses how to develop a practical and efficient LC-HTA process by utilizing the LC-HTA framework discussed above and by providing high-level worked examples of HTA response to accelerated regulatory approval and iterative innovation.
